# Visual Prognosis after Explantation of Small-Aperture Corneal Inlays in Presbyopic Eyes: A Case Series

**Published:** 2019

**Authors:** Majid Moshirfar, David F. Skanchy, David B. Rosen, Madeline B. Heiland, Harry Y. Liu, Benjamin Buckner, Aaron T. Gomez, Yasmyne C. Ronquillo, Tim Melton, Phillip C. Jr Hoopes

**Affiliations:** 1 John A. Moran Eye Center, Department of Ophthalmology and Visual Sciences, School of Medicine, University of Utah, Salt Lake City, UT, USA; 2 Utah Lions Eye Bank, Murray, UT, USA; 3 Hoopes Durrie Rivera Research Center, Hoopes Vision, Draper, UT, USA; 4 Department of Ophthalmology and Visual Sciences, W.K. Kellogg Eye Center, Medical School, University of Michigan, Ann Arbor, Michigan, USA; 5 College of Medicine-Phoenix, the University of Arizona, Phoenix, AZ, USA; 6 Health Science Center at Houston, McGovern Medical School, The University of Texas, Houston, TX, USA; 7 Rio Grande Valley School of Medicine, University of Texas, Edinburg, TX, USA

**Keywords:** Cornea, Explantation, KAMRA, Small Aperture Inlay, Presbyopia

## Abstract

The purpose of this study was to report visual prognosis after explantation of a small-aperture corneal inlay used for the treatment of presbyopia. This is a retrospective case series conducted at a single site in Draper, Utah, USA (Hoopes Vision). Medical records of 176 patients who had received a small-aperture corneal inlay (KAMRA™, AcuFocus Inc., Irvine, CA, USA) were reviewed. Patients who had undergone explantation of the device were identified. Uncorrected distance visual acuity (UDVA), uncorrected near visual acuity (UNVA), corrected distance visual acuity (CDVA), and manifest refraction spherical equivalent (MRSE) were measured pre-implantation, post-implantation, pre-explantation, and post-explantation of the inlay. Ten eyes from ten patients were included in this study. The explantation rate was 5.7% over 31 months, with blurry vision as the most common complaint. After explantation, six patients achieved pre-implantation UDVA, and six achieved pre-implantation UNVA. Eight of nine patients who underwent final manifest refraction achieved pre-operative CDVA. All patients had residual donut-shaped corneal haze in the stroma at the previous position of the inlay. All patients experienced improvement in haze with 20% experiencing complete resolution. The degree of stromal haze was not related to the duration of implantation. Of the subset of patients who underwent explantation of their small-aperture corneal inlay, there was persistent loss of CDVA in 10%. The majority of patients experienced some level of residual stromal haze, which may contribute to deficits in UNVA and CDVA in few patients. A hyperopic shift induced by the corneal inlay may contribute to the blurry vision these patients experienced; there was a reduction of this shift post-explantation. While this device is removable, patients should expect some post-explantation changes such as residual haze with a small subset experiencing persistent deficits in CDVA.

## INTRODUCTION

The KAMRA™ inlay (AcuFocus Inc., Irvine, CA, USA) is an implantable ring-shaped device for the treatment of presbyopia. It is designed for the non-dominant eye and approved by the U.S. Food and Drug Administration (FDA) and European Commission. The inlay is 3.8mm in diameter with a 1.6 mm diameter hole in the center and is made of Polyvinylidene Fluoride with carbon black pigment. It is implanted into a corneal pocket created by a femtosecond laser. Using the principle of pinhole optics, it increases the depth of focus and improves near vision without compromising the distance acuity [1]. Recent studies have demonstrated both the safety and efficacy of the device [2-4]. While the inlay is typically well tolerated, there are cases of patients requesting removal secondary to visual disturbances, unsatisfactory results, or other adverse outcomes that can accompany corneal and refractive surgeries [2-5]. In the initial FDA trial of 508 eyes, the explantation rate was 7.1% and 8.7% at 24 and 36 months respectively [1]. As this is a relatively new device, current literature on the visual outcomes following explantation of the inlay is lacking. We report on the visual outcomes of ten patients who underwent explantation of the KAMRA™ inlay. 

## METHODS

This study is a retrospective case series of patients who underwent explantation of the KAMRA™, small aperture corneal inlay. Between May 2015 and August 2018, 176 patients received the inlay at the Eye Surg of Utah, Hoopes Vision, United States. The medical records of patients who underwent KAMRA implantation were reviewed to identify all patients who had undergone explantation of this device from May 2015 to December 2018. The device removed in this study was the third-generation KAMRA™ corneal inlay (ACI7000PDT). Data on Snellen uncorrected distance visual acuity (UDVA), uncorrected near visual acuity (UNVA), corrected distance visual acuity (CDVA), and manifest refraction spherical equivalent (MRSE) were gathered at four time points: pre-implantation, post-implantation, pre-explantation, and post-explantation of the inlay. Approval was obtained from the Hoopes research committee, and informed consent was signed by each patient. All procedures adhered to the tenets of the Declaration of Helsinki. Data obtained from patients were expressed in mean, frequency, and percentage.

## RESULTS

**Figure 1:**
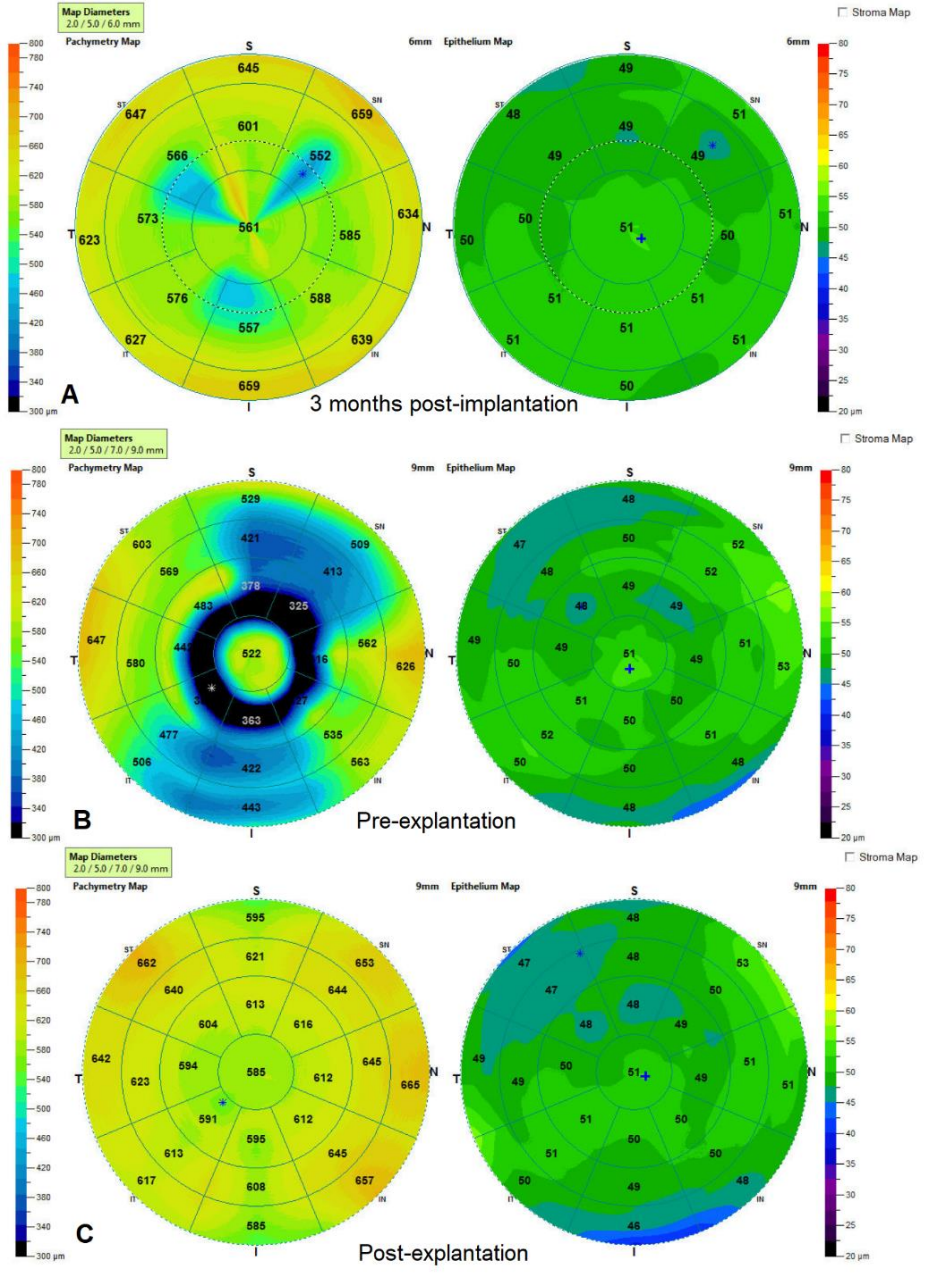
Anterior segment optical coherence tomography (OCT) imaging 3 months’ post-implant (A), pre-explantation (B), and post-explantation (C) of KAMRA inlay. The induced donut-shaped topographic change develops with time and normalizes after explantation. (Case 8)

Ten patients (5.7%) underwent explantation during a 31-month period secondary to complaints of “blurry vision” (6 patients), nighttime glare/difficulty driving at night (3 patients), discomfort (2 patients), unsatisfactory near vision (2 patients), and light sensitivity (1 patient). The average amount of time from implantation to explantation was 482 days (range 153-735); the time between explantation and most recent follow-up examination was 190 days on average (range 8-388). All eyes that underwent explantation had no other simultaneous corneal refractive surgery, i.e. LASIK. The average depth of implantation was 234 micrometers (range 205-250 µm). One patient underwent KAMRA repositioning prior to explantation, which did not alleviate symptoms. Fig. 1 show the changes in anterior segment optical coherence tomography (OCT) of stromal and epithelial maps before and after explantation.

**Figure 2:**
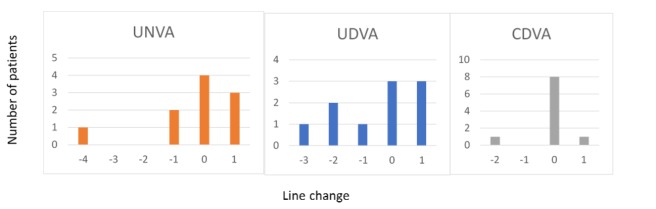
Visual Acuity Line Changes (Line Change) for UDVA, UNVA, and CDVA

Abbreviations: UDVA: Uncorrected Distance Visual Acuity; UNVA: Uncorrected Near Visual Acuity; CDVA: Corrected Distance Visual Acuity

Uncorrected distance visual acuity outcome: After explantation six of ten (60%) patients achieved pre-implantation UDVA (Table 1; Fig. 2). The average line change on the Snellen chart for UDVA between pre-implantation and post-explantation was -0.5 (Table 1). The median line change for UDVA was 0 (range -3 to +1).

Uncorrected near visual acuity outcome: Seven of ten (70%) patients achieved pre-implantation UNVA (Table 1); the average line change difference on UNVA was -0.3 (Table 1). The median line change for UNVA was 0 (range -4 to -1) (Table 1). 

Corrected distance visual acuity outcome: CDVA was retained by nine out of ten patients. One patient remained with a two-line loss greater than three months after explantation. 

**Figure 3:**
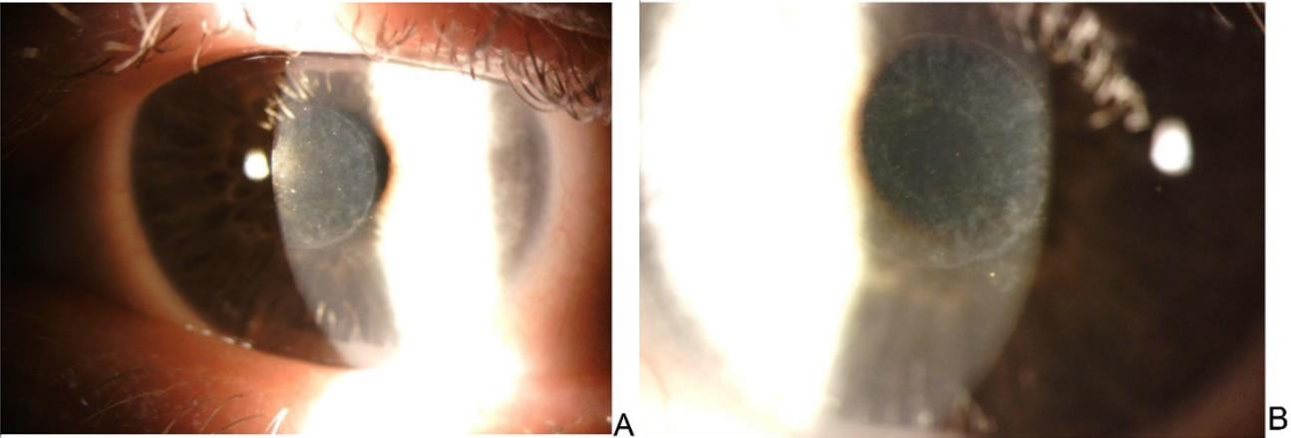
Corneal Haze Remaining at 1 Month (A) and 2.5 Months (B) after KAMRA Explantation in the Same Patient. The Degree of Corneal Haze Appears to Diminish with Time. (Case 10).

Haze: Fifty percent of patients had subjective complaint of haze, and all patients had some degree of corneal stromal haze on exam (Fig. 3). Two patients had complete objective resolution of haze (at one and three months respectively), while all other patients had residual stromal haze (up to 1.5 years after explantation). There was no correlation between the duration of implantation and the amount of stromal haze after explantation. 

Manifest refraction spherical equivalent: Nine patients completed final manifest refraction, and eight of the nine patients achieved pre-operative CDVA (Table 1). A hyperopic shift (ranging from +0.625 to +2.0 D) was noted in MRSE before explantation in 5 patients (50%) (Table 2). After explantation, this hyperopic shift completely or partially reversed in 4 of 5 patients.

## DISCUSSION

The results of this study demonstrate the removability and relative reversibility of the KAMRA corneal inlay. After explantation, most patients can expect to trend toward pre-implantation visual acuities. However, there is a risk that they may not achieve baseline acuity. In addition, patients can expect an improvement in haze or hyperopic shift, but these changes may be persistent after inlay removal. 

The KAMRA inlay had over 20,000 implantations during the first year of its release [6]. However, there have been few reports on inlay explantation and subsequent outcomes. The rate of KAMRA corneal inlay explantation in the literature ranges from 1.5-10% [1, 4, 7, 8]. In the original FDA trial, the vast majority of explantations were due to refractive shifts or dissatisfaction with visual outcomes [1]. Yilmaz et al. reported explantation secondary to either refractive shifts or flap complications [7]. Other studies reported explantation most often secondary to poor acuity or haze [4, 8, 9]. Other complaints may include “blurry vision”, decreased night vision, night glare, starbursts, halo, photophobia, and unsatisfactory near visual acuity [1, 10]. Our institution had an explant rate of 5.7% over approximately 2.5 years. In our series, 60% of patients who requested explantation complained of “blurry vision.” The term blurry vision is somewhat ambiguous, and the exact nature of what the term means to each patient varies. Blurry vision could be due to distortions from corneal haze, inlay induced hyperopic shift, irregular astigmatism, or a combination of these factors.

**Table 1:** Comparison of Visual Acuity from Pre-implantation to Most Recent Exam Post-explantation. Mean Values for UDVA and CDVA are Preserved, While There is a Slight Decrease in UNVA Post-explantation. Snellen Line Change is the Difference between Pre-implantation and Post-explantation

	Pre-implantation			Pre-explantation			Post-explantation			Snellen line change		
Case	**UDVA**	**UNVA**	**CDVA**	**UDVA**	**UNVA**	**CDVA**	**UDVA**	**UNVA**	**CDVA**	**UDVA**	**UNVA**	**CDVA**
1*	20/20	20/50	20/20	20/25	20/40	20/20	20/30	20/50	20/20	-2	0	0
2*	20/30	20/32	20/20	20/200	20/40	20/20	20/40	20/25	20/20	-1	1	0
3**	20/40	20/63	20/20	20/50	20/63	20/20	20/30	20/80	20/20	1	-1	0
4*	20/15	20/50	20/20	20/25	20/50	20/25	20/25	20/50	20/20	-2	0	0
5	20/20	20/40	20/20	20/25	20/80	20/20	20/20	20/40	20/20	0	0	0
6	20/20	20/40	20/20	20/20	20/50	20/20	20/20	20/40	20/20	0	0	0
7	20/20	20/50	20/20	20/40	20/50	20/20	20/15	20/40	20/15	1	1	1
8**	20/25	20/50	20/20	20/30	20/63	20/20	20/25	20/63	20/20	0	-1	0
9**	20/25	20/40	20/20	20/40	20/50	20/20	20/20	20/100	20/20	1	-4	0
10‡	20/20	20/40	20/20	20/40	20/25	20/30	20/40	20/32	20/30	-3	1	-2
Mean	20/25	20/40	20/20	20/40	20/50	20/ 20	20/25	20/50	20 /20	-0.5	-0.3	-0.1

Abbreviations: UDVA: Uncorrected Distance Visual Acuity; UNVA: Uncorrected Near Visual Acuity; CDVA: Corrected Distance Visual Acuity

*: post-explantation loss of UDVA. **: post-explantation loss of UNVA. ‡: post-explantation loss of CDVA and UDVA

The data suggests that the majority of patients can expect to return to similar pre-implant values for UNVA and UDVA (Table 1; Fig. 2). While most patients regained pre-implantation CDVA, there is a risk that a patient may never regain their pre-operative CDVA. A hyperopic shift induced by the corneal inlay may contribute to the blurry vision. This shift may resolve either completely or partially after explantation. Complete resolution of hyperopia was seen in two of the patients with hyperopic shift (Table 2). Persistent residual corneal haze in the corneal stroma was common but did not result in loss of CDVA. This haze decreased over time (Fig. 3). The exact location of the haze (anterior or posterior) is difficult to assess. In the future, densitometry could aid in more specific characterization of the haze. Additionally, the implant induces a donut-shaped change in corneal epithelium (Fig. 1B). Topographical changes in the corneal epithelium appear to progress with the amount of time the inlay is in place (Fig. 1). However, this topographical change became more uniform after device explantation (Fig. 1C). This observation further reinforces the relative reversibility of the effects of the inlay. 

One limitation of this study is the relatively small sample size of patients who underwent explantation. Another limitation was the non-uniformity in follow up times. Despite these limitations, this study reports a wide variety of effects seen during inlay implantation and demonstrates trends of what patients may expect after explantation. Future research with a larger cohort and more consistent long-term follow up could help further characterize visual prognosis after KAMRA removal.

**Table 2:** Comparison of Manifest Refraction Spherical Equivalent (MRSE) From Pre-implantation to Post-explantation of KAMRA inlay. Five Patients Experienced a Hyperopic Shift Post-implantation, Which Either Completely or Partially Resolved After Explantation

	Pre-implantation	Pre-explantation	Post-explantation
Case	**MRSE**	**MRSE**	**MRSE**
1	-0.25	-0.75	-0.375
2	-0.75	-1.625	-1.125
3	-0.75	-0.75	+0.25
4†	-0.125	+0.625	-0.25
5†	-0.375	+0.625	-0.5
6	0	0	-0.375
7†	-0.375	+1.625	+0.125
8†	-0.5	+0.75	+0.375
9†	-0.75	-0.125	NA
10	-0.375	-1.25	-0.75
Mean	-0.425	-0.088	-0.292

†Patients with hyperopic shift pre-explantation. NA: Not Available.

## CONCLUSION

The results of this case series demonstrate the removability of the inlay and relative reversibility of the effects of the KAMRA inlay. On average, patients lost a few letters of UDVA and UNVA post-explantation. However, two patients had two or more lines lost on either CDVA or UNVA. In light of these findings, consideration should be taken regarding potential long-term consequences of explantation in a patient who does not tolerate the inlay. Patients should be aware that they may not return to their pre-implantation visual acuity and that some degree of residual haze post-explantation is likely.

## DISCLOSURE

Ethical issues have been completely observed by the authors. All named authors meet the International Committee of Medical Journal Editors (ICMJE) criteria for authorship of this manuscript, take responsibility for the integrity of the work as a whole, and have given final approval for the version to be published. No conflict of interest has been presented. Phillip C Hoopes Jr, MD is a consultant for CorneaGen.

## Funding/Support:

Research to Prevent Blindness, NY, USA
